# Effects of Polymorphisms in the SjSP-13 Gene of *Schistosoma japonicum* on Its Diagnostic Efficacy and Immunogenicity

**DOI:** 10.3389/fmicb.2018.01695

**Published:** 2018-07-25

**Authors:** Xindong Xu, Xiaobing Cui, Liufang Zhu, Zhengli Li, Yuanbin Zhang, Li Ma, Weiqing Pan

**Affiliations:** ^1^Institute for Infectious Diseases and Vaccine Development, Tongji University School of Medicine, Shanghai, China; ^2^Department of Tropical Infectious Diseases, Second Military Medical University, Shanghai, China

**Keywords:** schistosomiasis japonica, diagnosis, gene polymorphism, SjSP-13, biomarker

## Abstract

Schistosomiasis japonica is one of the most prevalent parasitic diseases in China. The scarcity of effective diagnostic tools is a major factor that contributes to the high prevalence of schistosomiasis japonica. SjSP-13 is a promising serological diagnostic biomarker of the disease. However, it is unclear whether polymorphisms in SjSP-13 affect its diagnostic efficacy and immunogenicity. Here, we found the SjSP-13 gene was highly polymorphic, and all the alleles of the gene were clustered into two clades, clade A and B. SjSP-13.6 and SjSP-13.25, the representative alleles of clade A and B, were produced in *Escherichia coli*. The diagnostic value of SjSP-13.6 (AUC = 0.983 ± 0.006), was found to be similar to the SjSP-13.25 (AUC = 0.973 ± 0.009) by receiver operating characteristic (ROC) analysis. SjSP-13.6 and SjSP-13.25 have the same specificity (96.7%), while the sensitivity of SjSP-13.6 (90.4%) is slightly but not significantly higher than SjSP-13.25 (85.2%). The combination use of the two alleles (SjSP-13.6/25) didn’t increase the diagnostic performance of SjSP-13 as the AUC value of SjSP-13.6/25 is 0.977 ± 0.009, lower than individual SjSP-13.6 (AUC = 0.983 ± 0.006). In addition, we found the immunogenicity of clade A alleles is significantly higher than clade B in *Schistosoma japonicum* naturally infected animals and patients, as the mean antibody levels of SjSP-13.6 was significantly higher than SjSP-13.25. We conclude that polymorphisms of the SjSP-13 gene should not affect its diagnostic efficacy, and it is not necessary to combine the alleles of the two clades for diagnosis of schistosomiasis.

## Introduction

Schistosomiasis is a major parasitic disease that affects more than 200 million people in 70 developing countries, and it causes the loss of at least 70 million disability-adjusted life years (Steinmann et al., 2006; Colley et al., 2014). Schistosomiasis japonica is mainly found in China and to a lesser extent in the Philippines (Rollinson et al., 2013; Xu et al., 2016). In China, about 12 million people were infected in the 1950s. After decades, schistosomiasis has been largely controlled in China through widespread treatment with the anthelmintic drug praziquantel and large-scale environmental campaigns to eradicate snails, which are the intermediate host of the parasites (Zhou et al., 2005, 2007; Xu et al., 2016). However, in China, control of schistosomiasis is particularly challenging because of the widespread distribution of the snail hosts and wide range of domestic and wild mammals that act as reservoirs for human infection (Minggang and Zheng, 1999; Gray et al., 2009). Moreover, there is no licensed vaccine for human or animal use (El Ridi et al., 2015; Ricciardi and Ndao, 2015; Merrifield et al., 2016). Thus, schistosomiasis japonica remains one of the most important public health problems in China.

A major factor that contributes to the disease prevalence of schistosomiasis japonica is the scarcity of effective diagnostic tools for detecting schistosome infections, particularly low-intensity infections (Zhou et al., 2005, 2007; Utzinger et al., 2015). Community diagnosis is also essential for identifying the target population for chemotherapy and evaluating the prevalence of schistosomiasis (Wu, 2002; Inobaya et al., 2018). In addition, current plans for the elimination of schistosomiasis highlight the importance of developing highly sensitive diagnostic tools for low-transmission environments because elimination of the source of the infection is a key strategy (Wang et al., 2008; Gray et al., 2010). However, the sensitivity of traditional direct parasitological techniques, such as the Kato-Katz method is quite poor. Patients with the moderate and light infection intensity were difficult to be diagnosed by Kato-Katz method, even using multiple fecal smears (Yu et al., 2007; Lin et al., 2008; Zhang et al., 2009). Development of immunodiagnostic techniques is always a priority for the diagnosis of schistosomiasis, and it was in fact integrated into the control programs in China as early as the 1980s (Wu, 2002). However, all the currently used immunodiagnostic techniques are based on crude antigens extracted from either eggs or worms (SEA or SWAP), resulting in a wide cross-reaction with antibodies to other flukes (Yu et al., 2007; Zhou et al., 2008). To improve specificity, it is essential to identify a single molecule marker instead of crude antigens (Wang and Hu, 2014).

In our previous study, we identified a novel protein marker, SjSP-13, through genome-wide screening of the proteins secreted by *Schistosoma japonicum* (Xu et al., 2014). SjSP-13 showed the adequate sensitivity required for identification of low-intensity infections and almost no cross-reactivity with the antibodies for other fluke infections. Use of a recombinant SjSP-13-based enzyme-linked immunosorbent assay (rSP13-ELISA) kit in a small-scale field study demonstrated a sixfold increase in the sensitivity for detection of *S. japonicum* infection. Moreover, the antibody to SjSP-13 decreased quickly after chemotherapy and about 75% patients became sero-negative six months after the drug treatment. Thus, rSP13-ELISA has some substantial advantages over other currently used methods in terms of sensitivity and specificity. However, we noted that the sequence of the SjSP-13 gene is polymorphic. It is unclear whether variations in this gene affect its diagnostic efficacy. Therefore, in this study, we analyzed the genetic polymorphisms of SjSP-13 and found that all the alleles could be differentiated into two distinct clades. Furthermore, we evaluated the diagnostic value of each clade.

## Materials and Methods

### Parasites and Animals

A field-collected isolate of *S. japonicum* from Guichi County, Anhui Province, China, was used in all the experiments. Twelve-week-old female New Zealand White rabbits and 6–8-week-old female BALB/c mice were obtained from SLAC Laboratory Animal Co., Ltd. of the Chinese Academy of Sciences of Shanghai. The parasites were maintained in *Oncomelania hupensis* snails and in the animal hosts. Animals were anesthetized by abdominal injection of 2% pelltobarbitalum natricum (50 mg/kg by body weight) and then fixed to wooden plates with rubber bands. The hairs on the abdomen were shaved using an animal clipper. The shaved part was then wetted with dechlorinated tap water. The mice and rabbits were infected with about 40 and 800 cercariae, respectively. All the procedures performed on the animals were conducted in accordance with and with the approval of the Internal Review Board of Tongji University School of Medicine.

### Characterization of SNPs in the ORF of SjSP-13

Adult worms were obtained by perfusion of the mesenteric vein of the rabbits. The total RNA was extracted from individual adult worms by using the TRIzol reagent (Invitrogen, United States). We searched for SNPs in the SjSP-13 coding region by sequencing RT-PCR products after cloning into the pUC19 vector. The cDNAs of 20 different worms were synthesized with the reverse transcriptase Superscript (TaKaRa, Japan) with oligo (dT) primers by using 0.1 μg of total RNA as the template. The complete 534 bp SjSP-13 open reading frame (ORF) was amplified using RT-PCR with an upstream primer, P1: 5′-GC GGA TCC ATG TTG AAA CGA TTA TTC ATA TTG-3′, and downstream primer, P2: 5′-CC GAA TTC TTA AGT GGT GAA TTG AAC TAG AAA C-3′. The RT-PCR products were sequenced after being inserted into the vectors. Three to 5 recombinant plasmids were selected for sequencing per worm. The DNA of isolates that showed clonal sequence variations (singletons or rare substitutions) was re-amplified and re-sequenced to confirm that the variations were genuine and not the result of incorporation errors by the Taq DNA polymerase. The raw sequence data were aligned using CLUSTAL X software (Larkin et al., 2007). The neighbor-joining tree of the SjSP-13 alleles and SmSP-13 (GenBank Accession No. AF029222) based on *p*, the proportion of amino acid difference, was generated using Mega 5 software (Tamura et al., 2011).

### Serum Collection

We collected 115 infected serum samples from villagers living in schistosomiasis-endemic areas, and the samples yielded positive results using the Kato-Katz method. The egg counts of these patients ranged from 8 to 320 EPG. We also collected 10 mouse sera and 6 rabbit sera at 6 weeks after the cercarial challenge. The sera of healthy humans and uninfected animals were used as the controls.

### Antigen Preparation

Two representative alleles of SjSP-13 (SjSP-13.6 and SjSP-13.25) were selected for gene expression. The coding sequences of the genes were optimized using *E. coli* codon. The synthesized fragments were inserted into the *E. coli* expression vector pET28a. Expression of the recombinant proteins was induced with 1 mM isopropyl-D-1-thiogalactopyranoside. The recombinant proteins were purified from the insoluble inclusion bodies with a hexahistidine tag. The purified antigens were re-natured in refolding buffer C7 (1 mM TCEP, 250 mM NaCl, 12.5 mM β-cyclodextrin, and 50 mM Tris-HCl [pH 8.5]). The purity of the purified proteins was determined using sodium dodecyl sulfate-polyacrylamide gel electrophoresis.

### ELISA

ELISA was performed to detect the IgG levels against SjSP-13.6, SjSP-13.25, and SjSP-13.6/25 combination (1:1). Briefly, 96-well plates (Corning, United States) were coated with 100 μL (per well) of 0.5 μg/mL antigens diluted in coating buffer for 16 h at 4°C. After blocking, sera (diluted 1:100) in blocking buffer were added to the wells. After washing, the plates were incubated with horseradish peroxidase-conjugated secondary antibodies (Abcam, United States) diluted (1:20,000) in blocking buffer. The plates were washed again, and 100 μL of TMB substrate solution was added to each well. The enzymatic reaction was stopped after 10 min of incubation at 37°C by adding 50 μL (per well) of 2 N H_2_SO_4_. The results were recorded as absorbance values at 450 nm with a microplate reader (Bio-rad, United States).

### Statistical Analysis

The SjSP-13 antibody levels in the human sera were determined using the ratio (R) of the OD_450__nm_ values to that of the negative reference serum. OD_450__nm_ and R values were expressed as mean ± SD. Differences of OD_450__nm_ and R values between groups were analyzed using Student’s *t*-test. The diagnostic accuracies of SjSP-13 antibody levels were assessed by receiver operating characteristic (ROC) curve analysis. The overall diagnostic performance of SjSP-13 is measured by calculating the area under the ROC curve (AUC). Differences of AUC were analyzed by *z*-test. The cutoff SjSP-13 antibody level was determined as *R* = 2.1, i.e., 2.1-folds of SjSP-13 antibody level of the negative reference serum. The sensitivity and specificity of different methods were calculated accordingly and compared using a Chi-square test. We used SPSS 12.0 software (IBM, United States) for all the statistical analyses, and a *p*-value < 0.05 was considered statistically significant.

## Results

### Sequence Polymorphism of the SjSP-13 Gene

**FIGURE 1:**
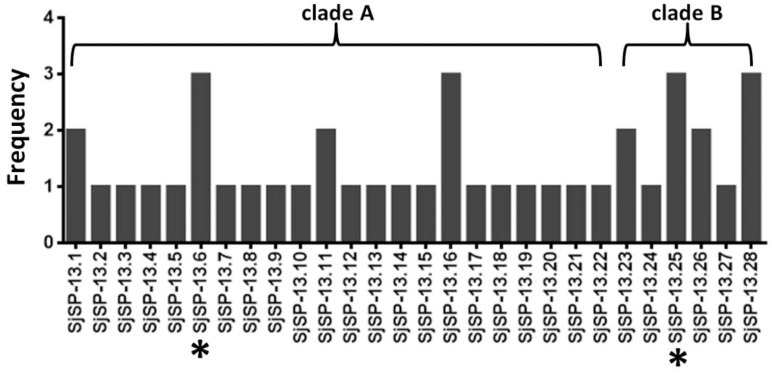
Allele frequency of 28 SjSP-13 alleles. ^∗^The representative alleles of SjSP-13 (SjSP-13.6 and SjSP-13.25) were selected for gene expression.

**FIGURE 2:**
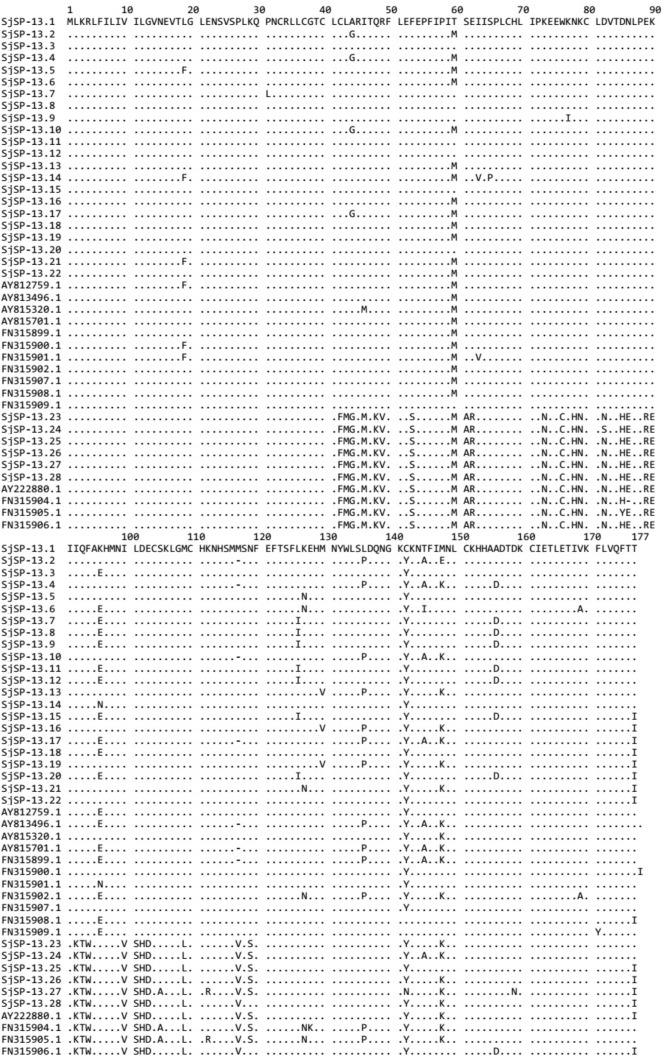
Alignment of amino acid sequences of SjSP-13 alleles. Numbers indicate the position of amino acid residues. Dots indicate the same amino acid residue as that present in SjSP-13.1.

**FIGURE 3:**
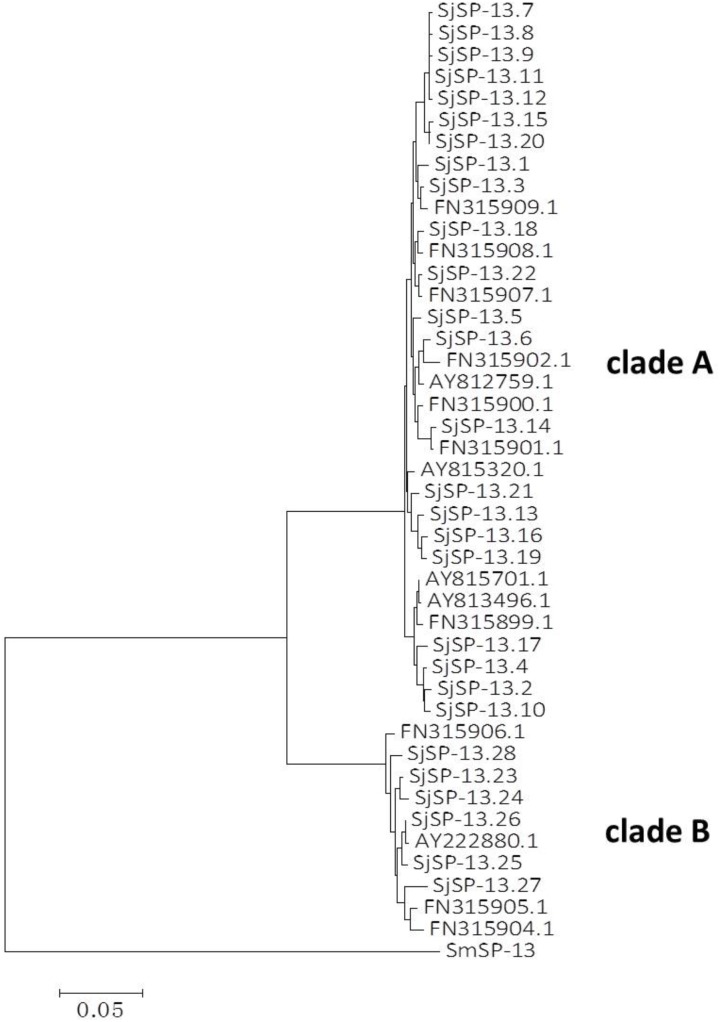
Neighbor-joining tree of SjSP-13 alleles and SmSP-13 (AF029222) generated using Mega 5.

We obtained a total of 40 allele sequences of the SjSP-13 gene from 20 individual worms derived from a field-isolate of *S. japonicum* cercariae through cloning and sequencing. From the 40 allele sequences, 28 different allelic forms (designated as SjSP-13.1 to SjSP-13.28) were identified. The frequency distribution of the alleles was variable, from 1 to 3 (**Figure 1**). In addition, we obtained 15 allelic forms of the SjSP-13 gene from GenBank. Thus, a total of 43 alleles of the gene were further analyzed by CLUSTA X (**Figure 2** and Supplementary Table 1). The relationships among the 43 alleles were established using neighbor-joining analysis (Mega 5). We found that all the alleles could be differentiated into two distinct clades, clades A and B (**Figure 3**). The DNA sequence of each clade was highly conserved (96.8 and 92.3% identity in clades A and B, respectively), while the sequence identity between the clades was only 78.3%. The orthologous gene SmSP-13 of *Schistosoma mansoni* was 61.0% identical to SjSP-13.

### SjSP-13 Allele-Specific Antibodies in Infected Animals and Patients

**FIGURE 4:**
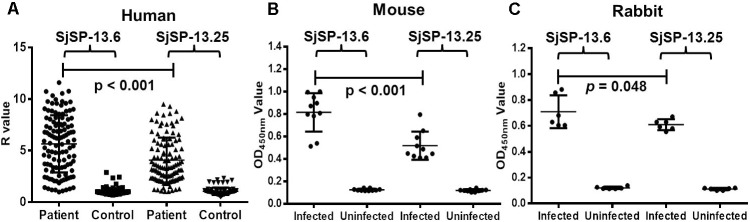
SjSP-13.6 and SjSP-13.25 specific antibody levels in schistosomiasis patients and infected animals. **(A)** SjSP-13.6 and SjSP-13.25 specific antibody levels in schistosomiasis patients (*N* = 115) and healthy controls (*N* = 90). **(B)** SjSP-13.6 and SjSP-13.25 specific antibody levels in *S. japonicum* infected mice (*N* = 10) and uninfected mice (*N* = 10). **(C)** SjSP-13.6 and SjSP-13.25 specific antibody levels in *S. japonicum* infected rabbits (*N* = 6) and uninfected rabbits (*N* = 6).

The recombinant proteins of SjSP-13.6 (representative allele for clade A) and SjSP-13.25 (representative allele for clade B) were produced in *E. coli* (Supplementary Figure 1). The levels of SjSP-13 allele-specific antibodies were examined by ELISA. The antibody levels of the two different alleles in *S. japonicum* infected mice, rabbits, and humans were significantly higher than that in uninfected controls (**Figures 4A–C**). Thus, alleles of both clade A and B were potential diagnostic biomarkers of schistosomiasis. In addition, we observed the mean level of SjSP-13.6 specific antibody was significantly higher than SjSP-13.25 in different hosts, indicating that the immunogenicity of the clade A alleles of SjSP-13 gene was higher than that of the clade B alleles.

### Diagnostic Efficacy of Different SjSP-13 Alleles

**FIGURE 5:**
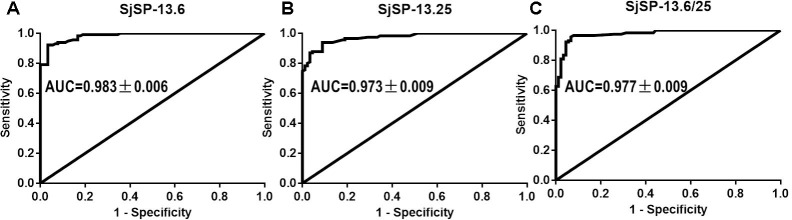
Receiver operating characteristic (ROC) analysis. ROC analysis was performed for SjSP-13.6 **(A)**, SjSP-13.25 **(B)**, and SjSP-13.6 combined with SjSP-13.25 **(C)**.

We collected 115 serum samples from schistosomiasis patients in Hunan and Jiangxi Provinces. 90 serum samples from healthy volunteers were collected as controls. The SjSP-13 antibody levels in the human sera were determined using the ratio (R) of the OD_450__nm_ values to that of the negative reference serum. To determine the diagnostic efficacy of different SjSP-13 alleles, we performed the ROC analysis for each allele and allele combination (**Figure 5**). The ROC analysis of the ELISA data showed that each allele had significant AUC. The AUC values of SjSP-13.6 and SjSP-13.25 are 0.983 ± 0.006 and 0.973 ± 0.009, respectively (**Figures 5A,B**). Here, the diagnostic cutoff values of SjSP-13.6 and SjSP-13.25 is determined as R = 2.1. We found SjSP-13.6 and SjSP-13.25 have the same specificity [96.7% (95%CI: 90.6–99.3%)], but the sensitivity of SjSP-13.6 [90.4% (95%CI: 81.5–93.4%)] is slightly but not significantly higher than SjSP-13.25 [85.2% (95%CI: 77.4–91.2%)] (**Table 1**).

**Table 1:** The diagnostic validity of SjSP-13.6, SjSP-13.25 and the combination of SjSP-13.6 with SjSP-13.25.

Antigens	AUC ± SE	*p-*value	Sensitivity (95%CI)	Specificity (95%CI)
SjSP-13.6	0.983 ± 0.006	<0.001	90.4% (81.5–93.4%)	96.7% (90.6–99.3%)
SjSP-13.25	0.973 ± 0.009	<0.001	85.2% (77.4–91.2%)	96.7% (90.6–99.3%)
SjSP-13.6/25	0.977 ± 0.009	<0.001	93.0% (86.8–97.0%)	93.3% (86.0–97.5%)

The combination use of the two alleles (SjSP-13.6/25) didn’t increase the diagnostic performance of SjSP-13 as the AUC value of SjSP-13.6/25 is 0.977 ± 0.009 (**Figure 5C**), lower than individual SjSP-13.6 (AUC = 0.983 ± 0.006) (**Figure 5A**). Although the combination use of SjSP-13.6 and SjSP-13.25 increased the sensitivity to 93.0% (95%CI: 86.8–97.0%), the difference is not significant when compared to individual SjSP-13.6 (**Table 1**). More importantly, the specificity of combination use of the SjSP-13.6 and SjSP-13.25 decreased to 93.3% (95%CI: 86.0–97.5%) (**Table 1**). These results indicated that the combination of multiple alleles did not improve the diagnostic performance of SjSP-13.

## Discussion

Identification of specific antigens that can be recognized by the host immune system is essential for immunodiagnosis and the development of vaccines (Loukas et al., 2011; Chen et al., 2016). The published genomes of *S. japonicum* and other species can help in the rapid identification of diagnostic markers (Berriman et al., 2009; The Schistosoma japonicum Genome Sequencing and Functional Analysis Consortium, 2009; Mitreva, 2012; Young et al., 2012). An increasing number of high-throughput immunological and proteomic studies have identified dozens of antigens as diagnostic markers or vaccine candidates, including tegumental and excretory-secretory proteins (Liu et al., 2009; Loukas et al., 2011; Driguez et al., 2016). Immunodiagnostic tools based on schistosome antigens are more sensitive than parasitological diagnostic tools and have thus become an attractive option. However, proteins with strong immunogenicity are often highly polymorphic to evade the selective pressure of the host immune system (Cupit et al., 2011; Zhang et al., 2011). Antigenic diversity could affect the diagnostic efficacy of antigen markers (Lee et al., 2006; Deme et al., 2014; Verma et al., 2015). Thus, it should be a prerequisite to check for genetic polymorphisms in diagnostic protein markers from a wide range of field isolates. In this study, we evaluated the polymorphism of SjSP-13 and impact of its genetic diversity on diagnostic efficacy.

SjSP-13 was first identified as a potential diagnostic protein marker by using a high-throughput glutathione *S*-transferase fusion protein array (Xu et al., 2014). This protein is a member of a multigene family of saposin-like proteins (Liu et al., 2016). In *S. japonicum*, this multigene family is present in the form of 15 members [SjSAPLP1 (SjSP-13) to SjSAPLP15]. Like SjSP-13, SjSAPLP4, and SjSAPLP5 are candidate diagnostic markers for schistosomiasis japonica. SjSP-13 is abundantly distributed on the surface and lumen of the esophageal and intestinal tracts of adult worms (Liu et al., 2016). In addition, SmSP-13, the homologous gene of SjSP-13 in *S. mansoni*, was found in worm vomitus(Hall et al., 2011). These results indicate that the continuous release of SjSP-13 from the intestinal tract directly exposes it to the host immune system and induces a strong immune response. The diagnostic efficacy of SjSP-13 has been proven in both infected animals and patients (Xu et al., 2014; Zhang et al., 2015; Liu et al., 2016). However, about 10% patients have been diagnosed as false-negative in different studies. When the sera of 302 subjects were tested by our group, the sensitivity of SjSP-13 was found to be 90.4% (Xu et al., 2014). In another small-scale test with 50 serum samples, the sensitivity of SjSP-13 was found to be 88% (Liu et al., 2016). In 9 patients from the Philippines, the sensitivity of SjSP-13 decreased to 66.7% (Cai et al., 2017). In this study, the sensitivity of SjSP-13 is also around 90% in 115 schistosomiasis patients. The false-negative results could be attributable to several reasons, such as the genetic diversity of SjSP-13 and low intensity of the infection.

To investigate whether the variations in the SjSP-13 gene is the cause of the false-negative result, we sequenced the ORFs of SjSP-13 in different parasites. We found that the sequence of the SjSP-13 gene is highly polymorphic and obtained at least 43 alleles. Unlike the Sj29 gene (Xu et al., 2012), another polymorphic gene of *S. japonicum*, no dominant allele was identified for the SjSP-13 gene; all 43 alleles could be clustered into clades A and B. It should be noted that this study evaluated the polymorphism of SjSP-13 based on a limited parasite population. We were not able to obtain schistosome-infected *O. hupensi*s snails from various endemic areas in China to release cercariae for establishment of other field strains for this purpose. Laboratory infection of snails with miracidium released from stool eggs may be an alternative method for establishing field strains in future studies.

The representative alleles of the clades, SjSP-13.6 and SjSP-13.25, were expressed in *E. coli*. The SjSP-13.6 allele was used for the detection of schistosome infection in the previous field study (Xu et al., 2014). In this study, we observed the similar diagnostic sensitivity and specificity of SjSP-13.6 and SjSP-13.25 alleles. SjSP-13.25 should have no additive effect on the diagnostic efficacy, as the combination of SjSP-13.6 and SjSP-13.25 didn’t increase the diagnostic performance of SjSP-13. However, the antibody levels for SjSP-13.6 and SjSP-13.25 in the infected animals and individuals were different. The mean level of the antibody specific for SjSP-13.6 was significantly higher than that for SjSP-13.25. These results indicated that the high immunogenicity of clade A alleles may be the results of the high expression levels of this clade in schistosome worms. However, the human host genetic background may also be a factor responsible for the difference in allele-specific antibodies.

Patient’s serum samples were collected from two major endemic areas, i.e., Dongting Lake in Hunan Province and Poyang Lake in Jiangxi Province. In China, schistosomiasis is mainly endemic to the areas around the lakes (Li-Juan et al., 2017). In 2016, a majority of the egg-positive samples were found in these two lake areas, accounting for more than 95% of the infected population in China. However, this parasite disease is also endemic to other areas in China, particularly in the mountainous area, such as Sichuan and Yunnan provinces where the parasite genetic background may be different from that in the lake districts. Therefore, additional investigations need to be conducted in a larger parasite population and different endemic areas to further confirm the sequence diversity and diagnostic efficacy of the SjSP-13-based method.

## Conclusion

The sequence of the SjSP-13 gene is highly polymorphic, and the alleles of the SjSP-13 gene can be divided into two clades. Polymorphisms of the SjSP-13 gene should not affect its diagnostic efficacy, and it is not necessary to combine the alleles of the two clades for diagnosis of schistosomiasis.

## Author Contributions

XX and WP conceived and designed the study. XX, XC, LZ, ZL, YZ, and LM performed the experiments and analyzed the data. XX and WP wrote the manuscript. All authors read and approved the final manuscript.

## Conflict of Interest Statement

The authors declare that the research was conducted in the absence of any commercial or financial relationships that could be construed as a potential conflict of interest.
