# Supplementary material for: Effects of Polymorphisms in the SjSP-13 Gene of Schistosoma japonicum on Its Diagnostic Efficacy and Immunogenicity
Source: Front Microbiol. 2018 Jul 25;9:1695. doi: 10.3389/fmicb.2018.01695 (PMC6094988; doi:10.3389/fmicb.2018.01695)
Supplement: Supplementary file 1 [file Data_Sheet_1.PDF]

Supplementary Table 1 Distribution of SjSP-13 alleles. Vertical numbers indicate the position of variable nuclear acids within the sequence. Dots indicate the same nuclear acids as is present in SjSP-13.1.

	Position of Nuclear Acids in SjSP-13 ORF									
	11111	1111111111	1111111111	1111222222	2222222222	2222222222	2222222233	3333333333	3333333333	4444444444
	1159900122	2233344444	5566777888	8999001112	3333444455	5666677777	7788889900	0000111233	4555567888	0002223344
Alleles	5870268456	7915824567	7858479145	7235143698	0127456936	8456803567	8912681801	2349235559	9014606128	0784593412
SjSP-13.1	ACAACCGTGC	CACAACAAGG	TTTTATCTGA	ATTTACCAAG	AAAGGACAGA	TTGAAGCTTC	AATCAACATC	TCGGCTCAAT	ATGAATCGGA	TTATGGACCA
SjSP-13.2	....	..G...G...	.....CT...	....	....	......T...	....	C	---.......	.C..A.G.AG
SjSP-13.3	....	....	....	....	....	......T...	....G.....	.T........	....	..T.A.....
SjSP-13.4	....	..G...G...	......T...	....	....	......T...	....	C	---.......	.C..A.G.A.
SjSP-13.5	..T.......	....	......T...	....	....	......T...	....	....	.......T..	....A.....
SjSP-13.6	....	....	......T...	.....T.G..	....	......T...	....G.....	.T........	.......T..	....A..T..
SjSP-13.7	....T.....	....	....	....	....	......T...	....G.....	.T.......C	......A...	....A.....
SjSP-13.8	...G......	....	....	....	....	......T...	....G.....	.T.......C	......A...	....A.....
SjSP-13.9	....	....	....	....						
SjSP-13.10	....	..G...G...	......T...	....	....	......T...	....	C	---.......	.C..A.G.A.
SjSP-13.11	....	....	....	....	....	......T...	....G.....	.T.......C	......A...	....A.....
SjSP-13.12	....	....	....	....	....	....	....G.....	.T.......C	......A...	....A.....
SjSP-13.13	....	....	......T...	....	....	....	....	....	.......A.G	.C..AA..A.
SjSP-13.14	..T.......	....	......T...							
SjSP-13.15	G.........	....	..C.......	....	....	......T...	....G.....	.T.......C	......A...	....A.....
SjSP-13.16	G.........	....	......T...	.....T.G..	....	......T...	....	....	.......A.G	.C..AA..A.
SjSP-13.17	G.........	..G...G...	......T...	....	....	......T...	....G.....	.T.......C	---.......	.C..A.G.A.
SjSP-13.18	G.........	....	......T...	....	....	......T...	....G.....	.T........	....	..T.A.....
SjSP-13.19	G.........	....	......T...	....	....	....	....	....	.......A.G	.C..AA..A.
SjSP-13.20	G.........	....	....	....	....	......T...	....G.....	.T.......C	......A...	....A.....
SjSP-13.21	G.T.......	....	......T...	....	....	......T...	....	....	.......T..	....AA..A.
SjSP-13.22	G.........	....	......T...	....	....	......T...	....	....	....	..T.A.....
AY812759.1	G.T.......	....	......T...	.....T.G..	....	......T...	....G.....	.T........	....	....A.....
AY813496.1	....	....	......T...	.......G..	....	......T...	....G.....	.T.......C	---.......	.C..A.G.A.
AY815320.1	G.........	....G.....	......T...	....	....	......T...	....	....	....	....A...A.
AY815701.1	....	....	......T...	....	....	......T...	....G.....	.T.......C	---.......	.C..A.G.A.
FN315899.1	G...T.....	....	......T...	....	....	......T...	....G.....	.T.......C	---.......	.C..A.G.A.
FN315900.1	G.T.......	....	......T...	.....T.G..	....	......T...	....	....	....	....A.....
FN315901.1	G.T.......	....	......T...							
FN315902.1	GT........	....	......T...	.....T.G..	....	......T...	....G.....	.T........	.......T..	.C..A...A.
FN315907.1	....	....	......T...	....	....	......T...	....	....	....	..T.A.....
FN315908.1	G.........	....	......T...	....	....	......T...	....G.....	.T........	....	....
FN315909.1	....	....	....	....	....	......T...	....G.....	.T........	....	....
SjSP-13.23	......AATT	AGGGGA.GTA	AGACG.TGAG	.C.ACT.TTC	.GCTA.TTCG	AAAGGATAGA	CTGG..TGAT	CACCT.GT..	G.AGGC....	....AA..A.
SjSP-13.24	......AATT	AGGGGA.GTA	AGACG.TGAG	.C.ACT.TTC	.GCTAGTTCG	AAAGGATAGA	CTGG..TGAT	CACC..GT..	G.AGGC....	....AAG.A.
SjSP-13.25	G.....AATT	AGGGGA.GTA	AGACG.TGAG	.C.ACT.TTC	.GCTA.TTCG	AAAGGATAGA	CTGG..TGAT	CACCT.GT..		
SjSP-13.26	G.....AATT	AGGGGA.GTA	AGACG.TGAG	.C.ACT.TTC	.GCTA.TTCG	AAAGGATAGA	CTGG..TGAT	CACCT.GT..	G.AGGC....	....AA..A.
SjSP-13.27	G.....AATT	AGGGGA.GTA	AGACG.TGAG	.C.ACT.TTC	.GCTA.TTCG	AAAGGATAGA	CTGG..TGAT	CACCTGTTG.	G.AGGC....	..TAA...A.
SjSP-13.28	G.....AATT	AGGGGA.GTA	AGACG.TGAG	.C.ACT.TTC	.GCTA.TTCG	AAAGGATAAA	CTGG..TGAT	CACCT.TT..	G.AG......	..T.AA..A.
AY222880.1	G.....AATT	AGGGGA.GTA	AGACG.TGAG	.C.ACT.TTC	.GCTA.TTCG	AAAGGATAGA	CTGG..TGAT	CACCT.GT..	G.AGGC....	....AA..A.
FN315904.1	GT....AATT	AGGGGA.GTA	AGACG.TGAG	.C.ACT.TTC	.GCTA.TTCT	AAAGGATAGA	CTGG..TGAT	CACCTGTT..	G.AGGC.TA.	CC..A...A.
FN315905.1	GT....AATT	AGGGGA.GTA	AGACG.TGAG	.C.ACT.TTC	.GCTA.TTTG	AAAGGATAGA	CTGG..TGAT	CACCTGTTG.	G.AGGC.T..	.C..A...A.
FN315906.1	G.....AATT	AGGGGA.GTA	AGACG.TGAG	.C.ACT.TTC	.GCTA.TTCG	AAAGGATAAA	CTGG..TGAT	CACCT.TT..		

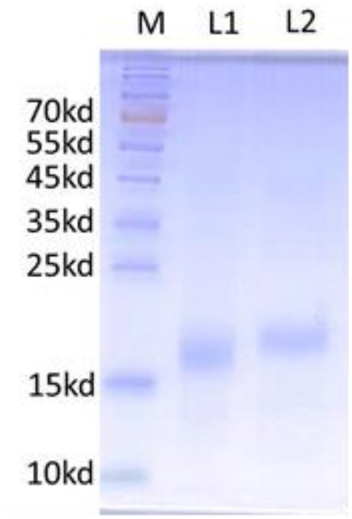

Supplementary Figure 1. SDS-PAGE analysis of purified recombinant antigens. M, Marker; L1, SjSP-13.25；L2, SjSP-13.6.
